# Whole-Genome Comparative and Pathogenicity Analysis of *Salmonella enterica* subsp. *enterica* Serovar Rissen

**DOI:** 10.1534/g3.120.401201

**Published:** 2020-05-01

**Authors:** Aiping Zhou, Jun Li, Zhihong Xu, Jinjing Ni, Jian Guo, Yu-Feng Yao, Wenjuan Wu

**Affiliations:** * Department of Laboratory Medicine, Shanghai East Hospital, Tongji University School of Medicine, Shanghai 200123, China,; ^†^ College of Biotechnology and Bioengineering, Zhejiang University of Technology, Hangzhou 310014, China, and; ^‡^ Laboratory of Bacterial Pathogenesis, Department of Microbiology and Immunology, Shanghai Jiao Tong University School of Medicine, Shanghai 200025, China

**Keywords:** *Salmonella enterica* serovar Rissen, host specificity, virulence, pathogenicity, genome

## Abstract

*Salmonella* are a type of bacteria known to cause food-borne illness. Their host range varies widely, and their susceptibility to the host determines its pathogenicity. *Salmonella enterica* serovar Rissen (*S*. Rissen) is a widely distributed serotype; however, its virulence and pathogenicity are poorly understood. In this study, the pathogenicity and antibiotic resistance of a representative *S*. Rissen isolate were investigated. The cell model results showed that *S*. Rissen preferred to replicate in human macrophage cells U937 compared to murine macrophage cells RAW264.7, suggesting that it has a level of host adaptability. Genome sequencing and comparison analysis revealed that the distribution and nonsynonymous single nucleotide polymorphisms of virulence factors in *S*. Rissen were similar to those in *S*. Typhi rather than to those in *S*. Typhimurium. Taken together, our results suggest that although *S*. Rissen is a common serotype distributed in swine herds, pork and chicken products, it has strong ability to infect humans.

*Salmonella enterica* is an important foodborne pathogen worldwide. Human infections with *Salmonella* normally occur through the consumption of contaminated food or water. Over 2600 different *Salmonella* serotypes are known (Edwards *et al.* 2002), and many can infect a broad spectrum of animal hosts, typically producing self-limiting gastroenteritis. However, a small number of serotypes exhibit narrow host specificity and show increased virulence in their specific hosts, which usually results in severe typhoid-like diseases. For example, *Salmonella enterica* serovar Typhi is host-restricted to humans, while *Salmonella enterica* serovar Typhimurium is frequently associated with diseases in numerous species, including humans, livestock, and rodents. Genome degradation and the elimination of genetic elements are commonly associated with host restriction (Toft and Andersson 2010; Spanò 2016). However, the molecular bases of *Salmonella*’s host restrictions are largely unknown (Dougan and Baker 2014; Spanò 2016). Therefore, the study of host-specific mechanism of *Salmonella* is of great significance for vaccine development and disease treatment.

In recent years, *Salmonella enterica* serovar Rissen (*S*. Rissen) has become a common serotype found in swine herds, pork and chicken products and gastrointestinal human patients throughout different regions of the world (Darwin and Miller 1999; Lee *et al.* 2016; Saito *et al.* 2016). In Thailand, *S*. Rissen is the most prevalent serotype isolate from swine and pork products (Dorn-In *et al.* 2009; Sanguankiat *et al.* 2010). Salmonellosis cases owing to *S*. Rissen infections in Europe (Garrido *et al.* 2015; Bonardi *et al.* 2016; Campos *et al.* 2016; Frasson *et al.* 2016) and America (Blondel *et al.* 2015; Molina *et al.* 2016) have been reported. Although there are many reports on *S*. Rissen-associated epidemics, to date, studies of its pathogenicity, virulence comparative genome analysis have been limited.

Here, we used mouse and cell models to evaluate the pathogenicity and virulence of a representative clinical *S*. Rissen strain, GJ0703, isolated from a diarrhetic patient with intestinal obstruction. The data revealed that *S*. Rissen possessed a host preference. To elucidate the molecular mechanism of *S*. Rissen’s host specificity and pathogenesis, its genome was sequenced and analyzed. This study increases the understanding of this serotype’s pathogenesis and provides a foundation for further studies on *Salmonella* virulence and host specificity.

## Materials and methods

### Salmonella isolation and serotyping

Two *S*. *enterica* strains, including a clinical *S*. Rissen strain (GJ0703) and the reference strain *S*. Typhimurium strain 14028S were used in this study. Strain GJ0703 was isolated from a patient’s stool in Shanghai, China in 2015. The samples were plated on selective MacConkey Agar (Shanghai Yihua Medical Technology Co., Ltd., Shanghai, China) and colonies from pure cultures were characterized using a VITEK 2 COMPACT automated system (bioMérieux, France). The isolates identified as *Salmonella* spp. were subjected to serotyping by slide agglutination, according to the Kaufmann-White classification using slide agglutination with O and H antigen-specific sera (Bio-Rad, Marnes-La Coquette, France; Staten Serum Institute, Copenhagen, Denmark; Sifin, Berlin, Germany) at the Shanghai Municipal Center for Disease Control and Prevention (Shanghai, China).

### Antimicrobial susceptibility testing

Antimicrobial susceptibility testing was applied according to standard protocols. Fourteen antimicrobials were used, including gentamicin, streptomycin, ampicillin, amoxicillin-clavulanic acid, cefoxitin, ceftiofur, ceftriaxone, azithromycin, chloramphenicol, nalidixic acid, ciprofloxacin, sulfisoxazole, trimethoprim-sulfamethoxazole, and tetracycline. MICs were determined by broth microdilution using the Sensititre plate CMV3AGNF (Sensititre, Thermo Fisher Scientific, USA). Resistance was defined using the CLSI-M100-S29 (2019), except for streptomycin (≥64 mg/l), azithromycin (≥32 mg/l) and ceftiofur (≥8 mg/l) for which there are no clinical breakpoints. The reference strain, *E. coli* ATCC 25922 was used as a quality control strain for determining the MIC of the antimicrobial agents.

### Animal experiments

All animal procedures were approved by Shanghai Jiao Tong University School of Medicine, and this study was carried out in strict accordance with the National Research Council Guide for Care and Use of Laboratory Animals [SYXK (Shanghai 2007-0025)]. Mice in the survival studies were monitored twice daily for weight, illness, and signs of discomfort, distress or pain. Mice that lost more than 20% of their initial weight were killed. At the end of the experiment, all mice were humanely killed. Euthanasia techniques (CO_2_ asphyxiation followed by cervical dislocation) followed the American Veterinary Medical Association Guidelines (2013). Animal experiments were performed as described previously (Ren *et al.* 2016). Inbred 8-week-old BALB/c mice were deprived of food and water for 4 h prior to administration of 20 mg of streptomycin per mouse by oral gavage. After 2 h, food and water were provided *ad libitum*. 20 h after streptomycin treatment, food and water were withdrawn again for 4 h. Afterward, 1.5 × 10^7^ CFU (colony-forming unit) bacteria in 200 μl PBS were administered by oral gavage, and control mice were given 200 μl PBS. Water and food were provided *ad libitum*. For intraperitoneal injection, 1.5 × 10^5^ CFU bacteria in 100 μl PBS were administered. There are 12 mice in each group. On day 2-post-infection, 6 mice from each group were killed. Spleens and livers from mice killed were collected, weighed, and then homogenized in PBS for CFU enumeration at designated time points. For the purposes of statistical analysis, bacterial counts were logarithmically transformed. The remaining 6 mice from each group were left until day 15-post-infection when the experiment was terminated. For recording survival rate, the number of live mice were counted twice daily.

### Cell infection assay

Human macrophage cell line U937 and murine macrophage-like cell line RAW264.7 were employed to determine the intracellular replication capability of *Salmonella* (Sang *et al.* 2017). RAW264.7 cells were obtained from the Cell Resource Center of the Shanghai Academy of Sciences, China. U937 cells were obtained from American Type Culture Collection (Manassas, VA, USA).

RAW264.7 cells were seeded at 2×10^5^ per well in 24-well plates and grown at 37° and 5% CO_2_ in DMEM supplemented with 10% fetal bovine serum (FBS), 100 units/ml penicillin G and 100 μg/ml streptomycin. The bacteria were diluted to achieve a multiplicity of infection (MOI) of 10, centrifuged at 400 g for 5 min at 25° to increase phagocytosis and incubated for 30 min at 37° in 5% CO_2_. After extensive washing with DMEM twice, infected cells were incubated in fresh tissue culture medium containing100 μg/ml gentamicin for the first 2 h post-infection or 15 μg/ml gentamicin for the remainder of the experiment. Infected cells were lysed at the desired post-infection time points with 0.1% (v/v) Triton X-100 in phosphate-buffered saline (PBS). The number of viable intracellular bacteria was determined by performing serial dilutions and plating. Bacterial growth was measured as the fold change in CFU /ml recovered from macrophages between two time points (Smith 1969).

U937 cells were cultivated in 24-well Nunc plates at a density of 5×10^4^ cells/ml/well. Prior to bacterial infection, the cells were cultured with 30 nM Phorbol myristate acetate for 2 d to differentiate them toward more mature macrophage-like cells. Before infection, the adherent cells were washed with PBS and then overlaid with pre-warmed RPMI 1640 supplemented with 10% FBS and 10mM HEPES. The cells were infected at a MOI of 100. After 2 h of infection, the cells were washed with PBS to remove non-adherent bacteria and overlaid with fresh RPMI 1640 containing 50 μg/ml gentamicin to kill extracellular bacteria. The *Salmonella*-infected cells were then incubated at 37° for 2 or 24 hr as indicated. To determine the number of living intracellular bacteria, the infected cells were lysed with 1% Triton X-100 in PBS at room temperature for 5-10 min, 100 μl of the lysate in ten-fold serial dilutions in PBS were added to LB agar plates, and the numbers of bacteria were reported as CFU.

### Whole-genome sequencing and annotation

Genomic DNA was isolated and purified using a DNeasy Kit (Qiagen, Valencia, CA, USA), and DNA concentrations were measured using a Qubit fluorometer (Life Technologies, Rockville, MD, USA). BGISEQ-500 was used for the GJ0703 sequencing at the Beijing Genomics Institute (BGI, Shenzhen, China).

Genomic DNA was randomly fragmented by Covaris. The fragmented genomic DNA was selected by magnetic beads to an average size of 200-400 bp. Fragment ends were repaired and then 3′ adenylated. Adaptors were ligated to the ends of these 3′ adenylated fragments. PCR process was to amplify fragments with adaptors from the previous step. PCR products were purified by the Magnetic beads. The double stranded PCR products were heat denatured and circularized by the splint oligo sequence. The single strand circle DNA (ssCir DNA) was formatted as the final library. The library was amplified with phi29 to make DNA nanoball (DNB) which have more than 300 copies of one molecule. The DNBs were load into the patterned nano-array and pair-end 100/150 bases reads were generated in the way of combinatorial Probe-Anchor Synthesis (cPAS).

The ORFs predicted by Glimmer3 (ccb.jhu.edu/software/glimmer) were annotated against the NCBI non-redundant protein databases using BLASTp algorithm-based searches. Subsequent analyses were performed using the Center for Genomic Epidemiology server (www.cbs.dtu.dk/services) as previously described (Thomsen *et al.* 2016). GJ0703 was classified into *Salmonella enterica* by using a highly accurate *k*-mer-driven species identification algorithm based on whole genome and phylogenetic relationship (Hasman *et al.* 2014; Larsen *et al.* 2014). A multilocus sequence typing analysis was executed using MLST 2.0 (cge.cbs.dtu.dk/services/MLST/) as previously described (Larsen *et al.* 2012). The replication origin of the GJ0703 genome was predicted by using Ori-Finder (Luo *et al.* 2019). Then, SPI Finder and VFDB (a web-based database of virulence factors, available at www.mgc.ac.cn/VFs/) (Chen *et al.* 2012) were used for the identification of *Salmonella* pathogenicity islands (SPIs) and virulence factors, respectively. VRprofile (bioinfo-mml.sjtu. edu.cn/VRprofile) (Li *et al.* 2018) was used to analyze the mobile genome, including plasmids, prophages, insertion sequence elements and associated gene cassettes, integrative conjugative elements (ICEs) and genomic islands (identity: 30%, e-value: 0.01 as cut-offs). Genetic organizations of SPIs were displayed and compared using linear schematics (Darling *et al.* 2004). The best matches to corresponding genes were determined using BLASTp, followed by verification using tBLASTn (identity: 40%, e-value: 0.001 as cut-offs).

### Genome analysis

First, the GJ0703 genome was systematically compared against seven other available Rissen genomes, in order to characterize the genetic difference between GJ0703 and other Rissen strains (GenBank Accession, 01-0479: MYYH00000000; 150: AHUI00000000; 2012K-0157: MYYQ00000000; BCW_2764: MYAA00000000; BCW_2765: MXZZ00000000; BCW_2766: MXZY00000000; BCW_2864: MXWY00000000). The core genes (conserved genes) of GJ0703 were determined based on 16 publicly available *Salmonella* genomes and their GenBank accession numbers are as follows: Agona str. SL 483 (CP001138), Choleraesuis str. SC-B67 (AE017220), Dublin str. CT_02021853 (CP001144), Enteritidis str. P125109 (AM933172), Gallinarum str. 287/91(AM933173), Heidelberg str. 41578 (CP004086), Newport str. SL254 (CP001113), Paratyphi A str. AKU_12601(FM200053), Paratyphi B str. SPB7 (CP000886), Paratyphi C str. RKS4594 (CP000857), Schwarzengrund str. CVM19633 (CP001127), Typhi str. CT18 (AL513382), Typhi str. Ty2 (AE014613) and Typhimurium str. 14028S (CP001363), Typhimurium str. LT2 (AE006468). All of the genomic data are available from NCBI (Supplementary Table S1). Core genes were identified using pan-genomes analysis pipeline (Zhao *et al.* 2012). A core genome was assigned using thresholds of 50% sequence identity and 50% sequence length coverage. Single nucleotide polymorphisms (SNPs) and indels of core genes were identified, respectively using the nucmer and mummer subprogram from the MUMmer3 software (Kurtz *et al.* 2004) against reference genomes.

Furthermore, conserved and specific genes among strains GJ0703, CT18 and 14028S were identified using all-against-all reciprocal BLASTp algorithm-based search and handy collation. Homologs were designated in cases of identity > 97% and e-value < 1E-10. Phylogeny based on these *Salmonella* genomes was calculated by CVTree3 (Zuo and Hao 2015), and evolutionary tree was generated by iTOL (Letunic and Bork 2019).

### Statistics

Statistical significance was determined with the unpaired Student’s *t*-test or Mann-Whitney test using Prism software (GraphPad Prism 5.0). All *P* values of 0.05 or less were considered significant and are referred to as such in the text.

### Data availability

The GJ0703 genomic sequences are available at GenBank (chromosome: ncbi.nlm.nih.gov/nuccore/CP043509, plasmid: ncbi.nlm.nih.gov/nuccore/CP043510). All the supplemental materials have been uploaded in GSA Figshare. Figure S1 shows the conserved and specific genes among strain GJ0703, CT18 and 14028S, alongside COG categories of 99 genes exclusively shared by GJ0703 and CT18. Figure S2 shows COG categories of the 928 genes carrying nsSNP variations shared by strains CT18 and GJ0703. Figure S3 shows the amino acid variation in McpC. Figure S4 shows the amino acid variation in IroC. Figure S5 shows the amino acid variation in IroE. Figure S6 shows SPI-1 and SPI-2 of *S. Rissen*. Figure S7 shows the genetic diversity and conservation of orthologous virulence factors in GJ0703, CT18 and 14028S. Figure S8 shows the amino acid variation in MgtC. Table S1 shows the genetic properties of reference strain genomes under study. Table S2 shows the examined antibiotic resistance profiles of strain GJ0703. Table S3 shows the virulence related genes shared by strains GJ0703 and CT18. Table S4 shows the 928 ORFs with the same nsSNPs shared by strains GJ0703 and CT18. Table S5 shows indels identified in GJ0703 and CT18 compared to 14028S, and indels shared by GJ0703 and CT18. Table S6 shows the comparative pathogenomics of *Salmonella* (total 17 genomes available). Supplemental material available at figshare: https://doi.org/10.25387/g3.11367824

## Results

### Salmonella isolation and genotyping

Strain GJ0703 was identified as *S*. Rissen by both the White-Kauffmann-Le Minor scheme using slide agglutination and SeqSero (www.denglab.info/SeqSero). The *in silico* multilocus sequence typing analysis showed that strain GJ0703 was ST469 by using the seven housekeeping gene types, which were 92 (*aroC*), 107 (*dnaN*), 79 (*hemD*), 156 (*hisD*), 64 (*purE*), 151 (*sucA*), and 87 (*thrA*). *S*. Rissen ST469 was reported as the epidemic-associated strain widespread in previous studies (Antunes *et al.* 2011; Campos *et al.* 2016; Mourão *et al.* 2016; Patchanee *et al.* 2017). Antimicrobial susceptibility testing indicated that GJ0703 strain was resistant to tetracycline, ampicillin, sulfisoxazole, trimethoprim-sulfamethoxazole and susceptible to all the rest antibiotics. Susceptibility data were shown in Supplementary Table S2.

### S. Rissen exhibits less virulence than S. Typhimurium in the mouse model

**Figure 1:**
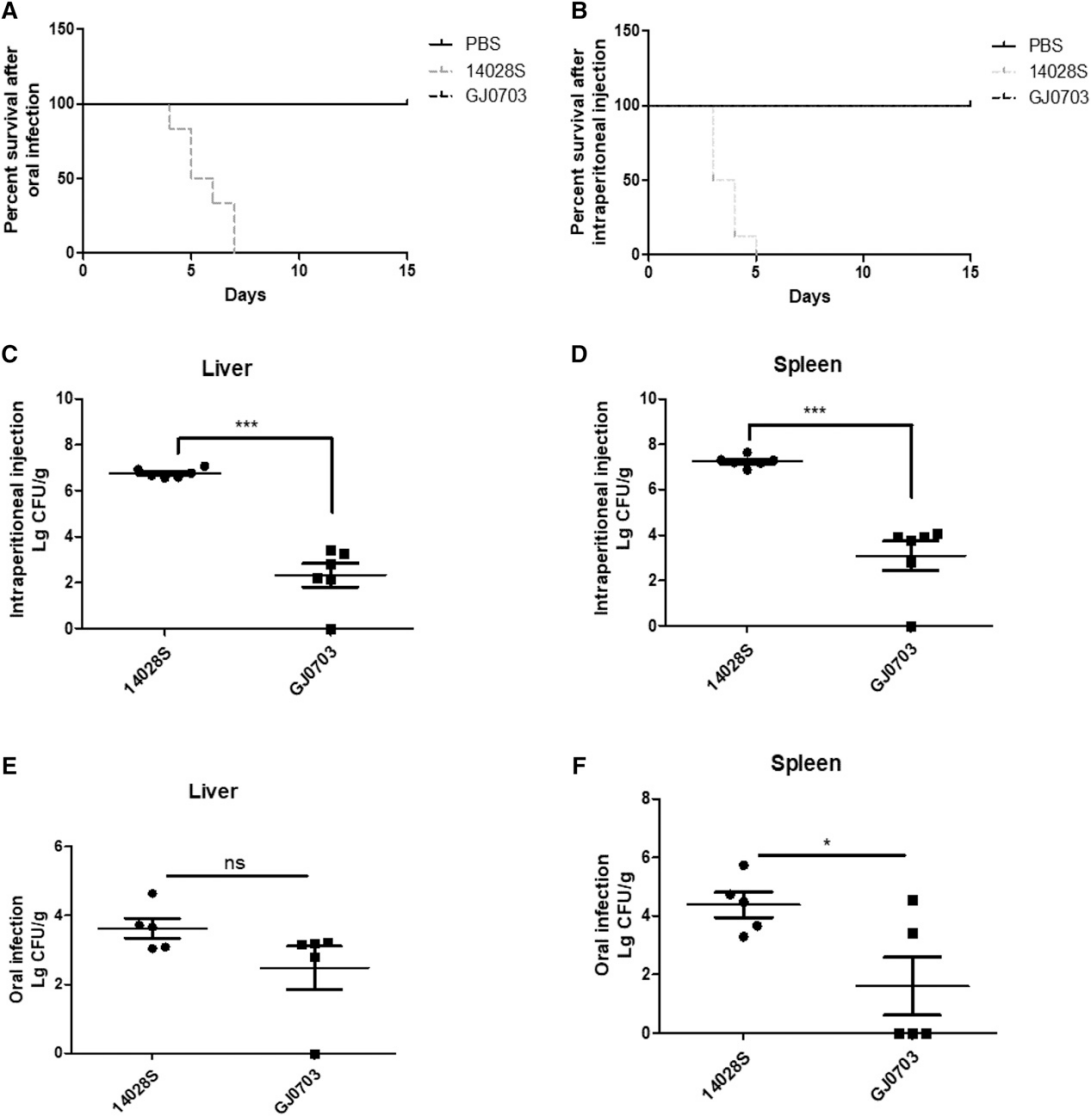
Rissen strain GJ0703 is hypovirulent in mice and causes an attenuated systemic infection. The survival of mice infected by oral gavage (A) or intraperitoneal injection (B). Bacterial burdens in livers (C) and spleens (D) of mice infected by intraperitoneal injection. Bacterial burdens in livers (E) and spleens (F) of mice infected by oral gavage. *, *P* < 0.05; ***, *P* < 0.001, (analysis of variance).

To evaluate the virulence of S. Rissen strain GJ0703 in mouse model, GJ0703 and 14028S were used to infect mice. First, mice were inoculated orally with 1.5 × 10^7^ CFU of *S*. Rissen strain GJ0703 or *S*. Typhimurium strain 14028S, and their mortality was monitored twice a day. Mice in the phosphate-buffered saline control group were alive and healthy throughout the study. Mice infected with strain 14028S were all dead or were killed from 4 d to 7 d after infection (Figure 1A). However, mice in the strain GJ0703 group showed a slight infection-associated morbidity, such as ruffling of fur and wasting on 2 d after infection, but recovered after day 3.

In general, an intraperitoneal infection is used to mimic the systemic phase of infection, bypassing the entry of the bacteria through epithelial cells into the gut. In addition, the invading bacteria depend on a set of invasion-related genes after oral infection that is not required for systemic virulence (Weintraub *et al.* 1997). Therefore, we repeated the survival assay using intraperitoneal infections with 1.5 × 10^5^ CFU of bacteria. Mice infected with strain 14028S began dying 3 d after intraperitoneal infection. In total, 3 of 7 mice died or were killed on d 3, and all of the mice were dead or were killed within 5 d after infection (Figure 1B). However, all of the mice infected with strain GJ0703 were alive for the whole experimental period. Thus, *S*. Rissen strain GJ0703 shows less virulence compared with *S*. Typhimurium in the mouse model.

To further determine the ability of *S*. Rissen strain GJ0703 to produce a systemic infection, the livers and spleens of mice were harvested 48 h after infection, and the bacterial burdens in the organs were recorded. The bacterial burdens of strain 14028S were greater than those of strain GJ0703 in the spleens and livers, regardless of the infection route (Figure 1C–F), indicating that the efficiency of *S*. Rissen to disseminate from either the gut or peritoneal cavity to systemic sites is lower than that of *S*. Typhimurium in the mouse model.

### S. Rissen prefers to replicate in human cells compared with murine cells

**Figure 2:**
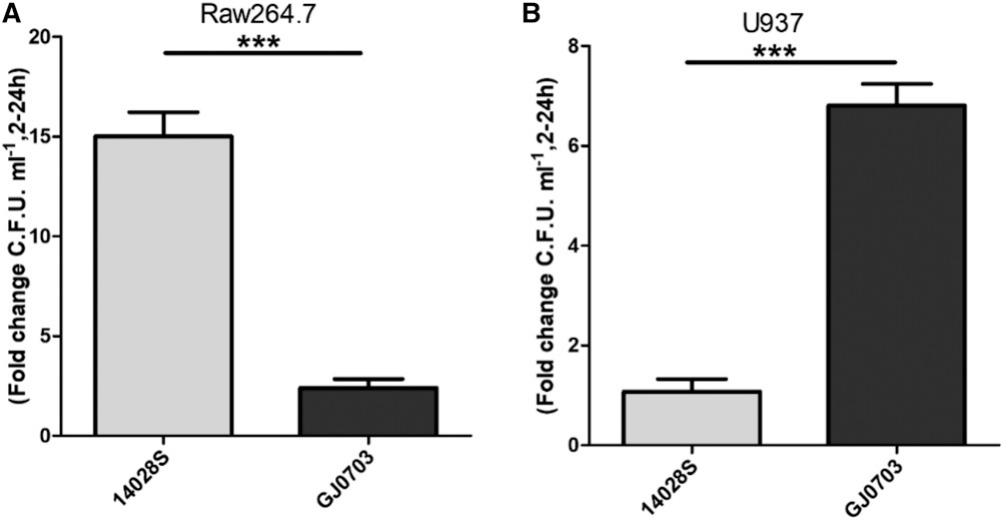
Evaluation of *S*. Rissen virulence in cell models. (A) Replication rates in RAW264.7 murine macrophage-like cells of strains 14028S and GJ0703. *Salmonella enterica* serovar Typhimurium 14028S and Rissen GJ0703 were grown with overnight shaking (250 rpm) in 5 ml LB medium at 37°C. RAW264.7 cells were infected at a multiplicity of infection of 10. Cells were lysed with 0.1% (v/v) Triton X-100 in phosphate-buffered saline (PBS) 2 and 24 h after infection. Lysates were diluted and plated on agar plates for bacterial colony counting. The replication rate was calculated as the fold change in bacteria recovered between 2 and 24 h after infection. (B) Replication rates in human macrophages U937 cells. For differentiation, U937 cells were stimulated with 30 nM phorbol myristate acetate for 2 d. The cells were infected at a MOI of 100, and the net growth between 2 and 24 h was calculated from the fold change in bacteria recovered at these time points. Error bars represent standard deviations from 3 independent experiments. ***, *P* < 0.001, (analysis of variance). Abbreviations: CFU, colony-forming unit; Lg, denary logarithm, or the base-10 logarithm; PBS, phosphate-buffered saline.

Intracellular replication in macrophages represents a major aspect of *Salmonella* virulence; therefore, we assessed the intracellular replication capabilities of strains GJ0703 and 14028S in the murine macrophage-like cell line RAW264.7 and human macrophage cell line U937. The intracellular bacterial number of strain GJ0703 increased only 2.4-fold after 24 h in RAW264.7 cells, while strain 14028S showed a 15-fold increase (Figure 2A). However, in U937 cells, the intracellular bacterial number of strain GJ0703 increased 7.4-fold after 24 h, while strain 14028S showed only a 1.4-fold increase (Figure 2B). These results suggest that *S*. Rissen has, to some extent, host specificity.

### Whole-genome sequencing and comparative analysis

To understand the host specificity of *S*. Rissen, we employed next generation sequencing to obtain the candidate genetic information of *S*. Rissen strain GJ0703. The assembly of strain GJ0703 included a chromosome of 4.93 Mbp in length with a GC content of 52.08%, and 1 plasmid replicon of 4,657 bp in length was found. Finally, 4,816 genes were predicted and 4,801 ORFs (protein coding sequences) were annotated, whereas only four genes were found in strain GJ0703 plasmid (coding for MbeA, MobC, macrophage stimulating factor and hypothetical protein, respectively).

**Figure 3:**
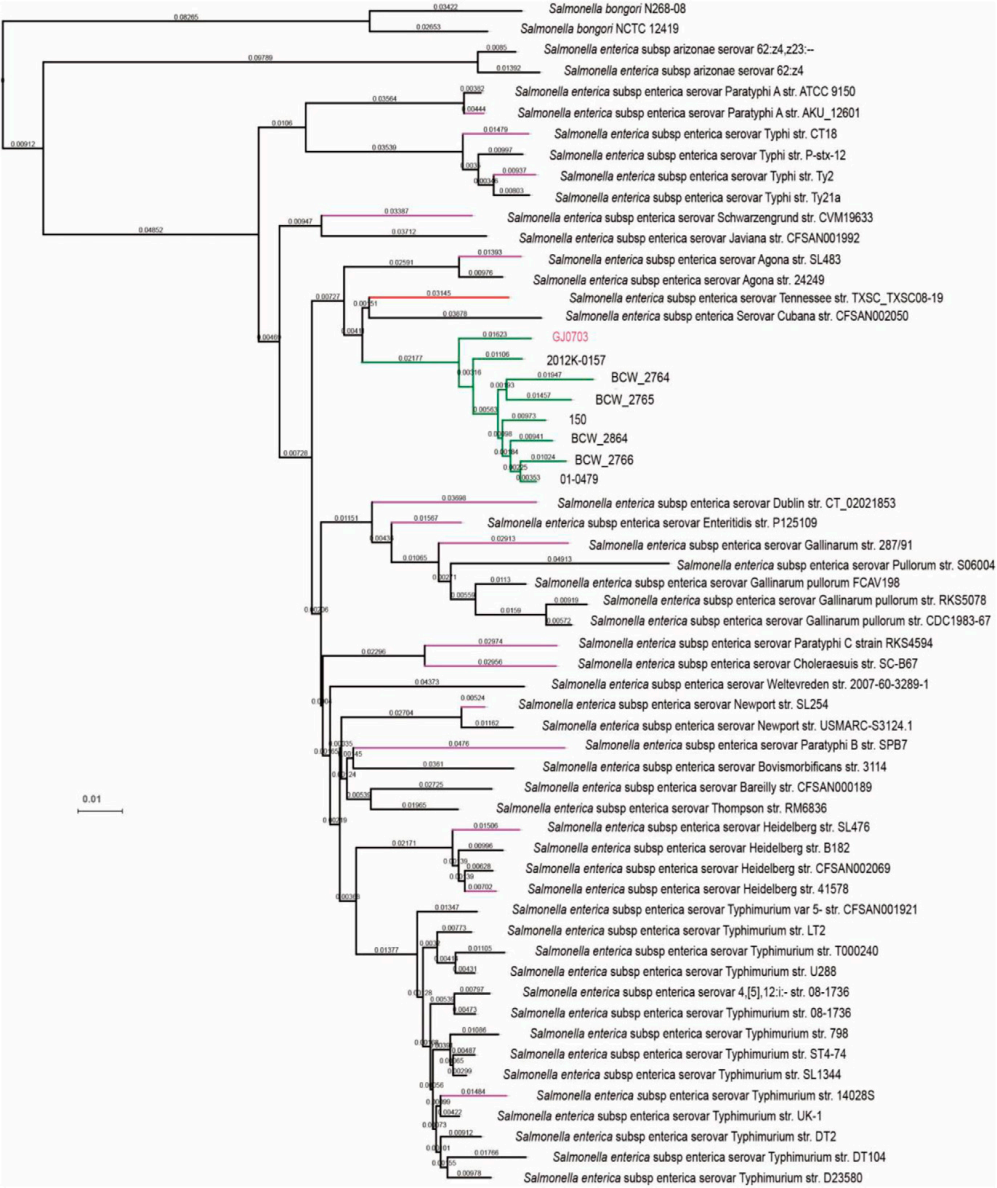
Phylogeny of *S. enteric*a Rissen GJ0703 and available *Salmonella* genomes. The GJ0703 genome and other Rissen strains constitute a compact group (green) in this tree and this clade is closely connected with serovar Tennessee strains. Sixteen reference *Salmonella* genomes listed in Table S1 was indicated by purple. Phylogenetic relationships were inferred by CVTree3 (available at tlife.fudan.edu.cn/cvtree), and evolutionary distances were denoted close to the phylogenetic clades.

We collected seven available genomes of serovar Rissen strains from NCBI archived data. The aforesaid strains’ genomes were deposited as draft sequences. To our knowledge, GJ0703 is the first closed genome for Rissen strains. In accordance to *Salmonella* phylogeny (Figure 3), GJ0703 presents a high coverage of the core genome with other Rissen genomes via comparative analysis based on single gene scale, including typical host-specialty related SPIs (*Salmonella* pathogenicity islands) and other common virulence factors in serotype Rissen. Furthermore, this observation verified the accurate serotype assignment for strain GJ0703.

**Figure 4:**
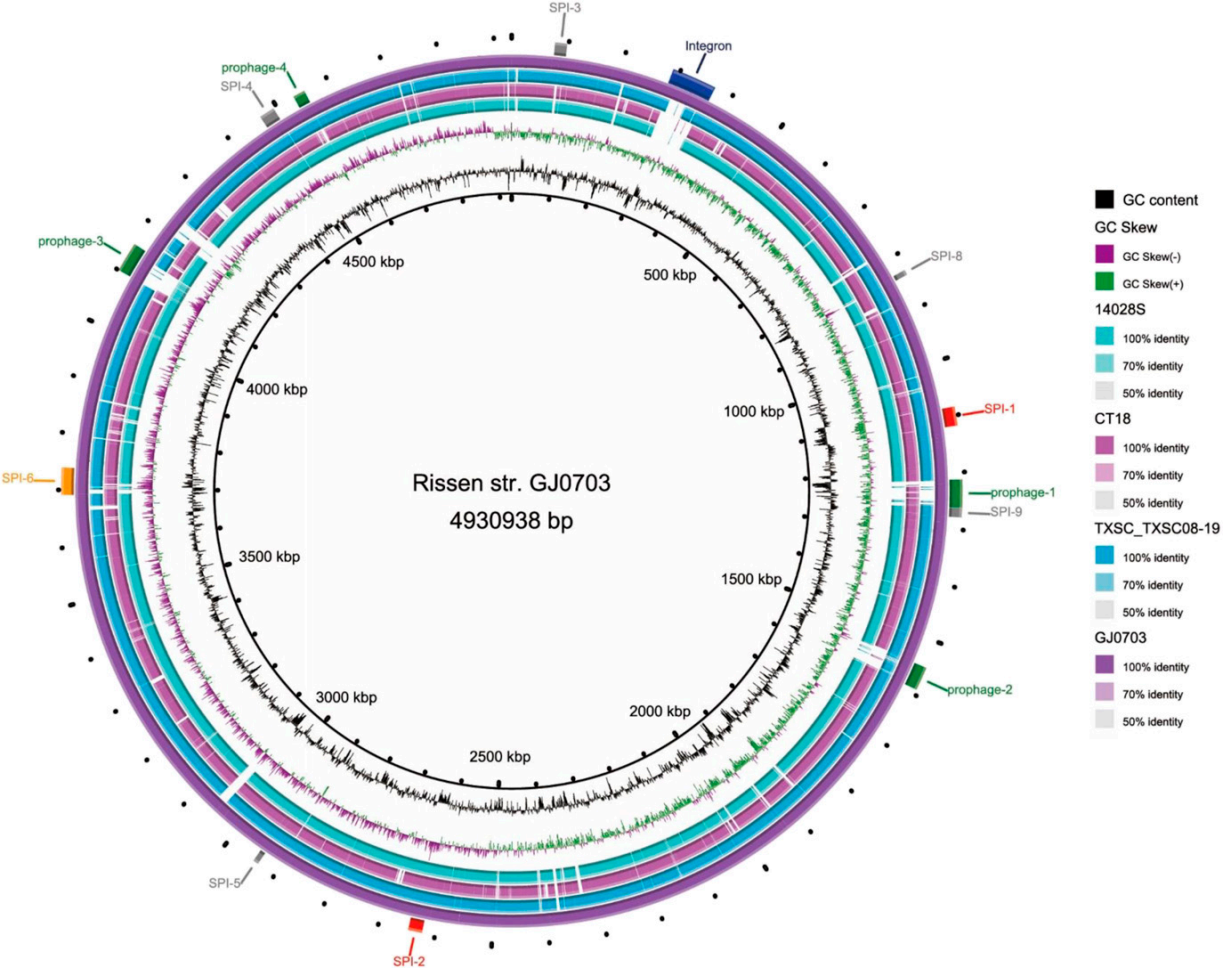
Circular schematics of the *S*. Rissen GJ0703 genome. From inner to outer circles: GC content, GC skew, homologs in strain 14028S, homologs in CT18, homologs in strain TXSC_TXSC08-19 and mobile genetic elements identified using VRprofile. Customized CGview and VRprofile analyses were performed using the *S*. Rissen genome to generate circular comparisons. The predicted *Salmonella* Pathogenicity Island (SPI) genes and other virulence determinants are respectively indicated and drawn to scale.

To investigate the pathogenicity of strain GJ0703, VRprofile was used to analyze the virulence related gene clusters in its genome. A total of 12 candidate regions were detected, including 2 type III secretion systems (T3SSs), 1 type VI secretion system (T6SS), 4 prophages and 1 integron (Figure 4). Of note, this integron-like region was not found in other reference genomes (Figure 4), featuring strain GJ0703 heterogeneity from horizontal genetic transfer.

We collected 16 representative serotype strains with apparent host preference from the *Salmonella* phylogeny, and we therefore used the pan-genomes analysis pipeline to determine the core genes along with *S*. Rissen strain GJ0703. Next, 2630 core genes were identified in these 17 *Salmonella* strains. S. Typhimurium is a representative serovar causing enteritis in a broad range of hosts and produces murine typhoid fever, while *S*. Typhi is a human-restricted serovar that causes human typhoid fever. Because *S*. Rissen showed a human host specificity, the genome of *S*. Rissen strain GJ0703 was compared with those of *S*. Typhimurium 14028S and *S*. Typhi CT18. The genome comparison showed that 3583 ORFs are shared in 14028S, CT18 and GJ0703. GJ0703 strain’s genome has 549 unique ORFs, and 99 ORFs are exclusively shared by CT18 and GJ0703. Most of these 99 ORFs were annotated to virulence and mobile genetic element related genes, including 12 genes associated with mobilome, prophages, transposons (Supplementary Fig. S1 and Table S3).

We then analyzed nonsynonymous SNPs (nsSNPs) and indels in core genes of strains GJ0703 and CT18 using strain 14028S as the reference genome. A total of 3419 nsSNPs distributed in 1368 ORFs were found in strain GJ0703 genome, and 4951 nsSNPs in 1767 ORFs were found in strain CT18’s genome. The genomes of strains GJ0703 and CT18 share 1622 nsSNPs distributed in 928 ORFs (Supplementary Table S4). The results of the core gene indel analysis are listed in Supplementary Table S5. In total, 30 of 57 indels in strain GJ0703 were also present in strain CT18.

COG classifications were conducted for those 928 ORFs with the same nsSNPs shared by strains GJ0703 and CT18, and most genes are associated with bacterial metabolism and pathogenesis, including Defense mechanisms [12] Intracellular trafficking, secretion, and vesicular transport [15], Secondary metabolites biosynthesis, transport and catabolism [20], Lipid transport and metabolism [24], Nucleotide transport and metabolism [28], Signal transduction mechanisms [39], Cell motility [29], Posttranslational modification, protein turnover, chaperones [40], Inorganic ion transport and metabolism [64], Energy production and conversion [65], Coenzyme transport and metabolism [66], Amino acid transport and metabolism [95], Cell wall/membrane/envelope biogenesis [81], Carbohydrate transport and metabolism [90] (Supplementary Fig. S2 and Table S4).

Several virulence genes harbored multiple nsSNPs. For example, there are 18 nsSNPs in *mcpC* (GJ0703GL000698), a member of the flagellar regulon involved in chemotaxis (Supplementary Fig. S3), 17 nsSNPs in *iroC* (GJ0703GL001198, ABC transporter ATP-binding protein/permease, involved in ferric uptake) (Supplementary Fig. S4), 4 nsSNP in *iroE* (GJ0703GL001196, Supplementary Fig. S5), 16 nsSNPs in *sifA* (GJ0703GL002776), 15 nsSNPs in *sseC* (GJ0703GL002598), 2 nsSNPs in *mgtC* (GJ0703GL000078), 2 nsSNPs in *fimH* (GJ0703GL003377) and 3 nsSNPs in *fimA* (GJ0703GL003381).

### Salmonella pathogenicity islands (SPIs)

Many *Salmonella* virulence factors, such as adhesins, invasins, and toxins are commonly clustered in certain regions of the chromosome known as SPIs (Santos *et al.* 2003). Eight SPIs, SPI-1 to 6, SPI-8 and SPI-9, were identified in *S*. Rissen strain GJ0703 genome (Figure 4). SPI-1 and SPI-2 are critical to *Salmonella* virulence, and a co-linear analysis showed that SPI-1 and SPI-2 of strain GJ0703 are highly homologous to those of *S*. Typhimurium and *S*. Typhi strains (Supplementary Fig. S6). Of note, *avrA* genes (coding for AvrA, T3SS effector) were both observed in strain CT18 (serotype Typhi) and strain GJ0703 (serotype Rissen). Furthermore, no *avrA* was found in strain LT2 (serotype Typhimurium).

**Figure 5:**
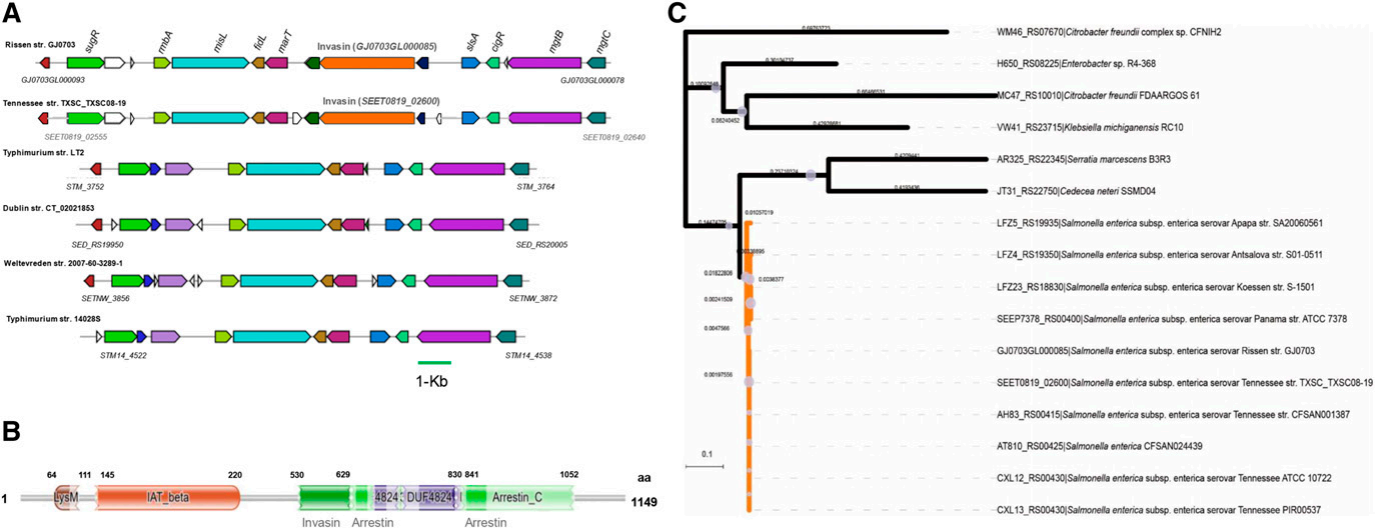
Comparative analysis of *S*. Rissen SPI-3. Schematic diagram of the genetic organization and invasin phylogeny of SPI-3. (A) An insertion fragment contains the invasion-associated genes. The SPI-3 homologs of *S*. Rissen were collinearly compared with the counterparts in the genomes of strains Typhimurium LT2, Dublin CT0202183, Tennessee TXSC_TXSC08-19 and Typhimurium 14028S. The genes of mosaic SPI-3 homologs identified in a variety of *Salmonella* genomes were color coordinated, and insertion genomic fragments were defined. (B) Conserved domains of this invasin predicted by Pfam (available at pfam.xfam.org, e-value ≤ 0.01). LysM: Lysin Motif, a protein domain found in a wide variety of extracellular proteins and receptors. IAT_beta: Inverse autotransporter beta-domain, a family of beta-barrel porin-like outer membrane proteins from enteropathogenic Gram-negative bacteria. DUF4824: the function of this family is unknown but DUF4824 is mainly found in *Pseudomonas* species. (C) Phylogenetic relationships of invasion homologs were inferred by using MEGA7 (available at megasoftware.net, NJ methods), and evolutionary distances and bootstrap values were denoted close to the phylogenetic clades. This gene was only found in *Salmonella enterica* serovar Tennessee strains and their closely-related isolates. Homologs of this invasin were predicted by BLASTp-based searches (threshold: identity ≥ 40%, e-value ≤ 0.01).

A novel insert fragment containing three ORFs was found in strain GJ0703 SPI-3 (Figure 5A). GJ0703GL000085 harbored a LysM superfamily domain, a small domain involved in peptidoglycan-binding, and an adhesin involved in diffuse adherence (AidA) superfamily domain, which is the adhesin of the bacterial autotransporter system (Figure 5B). It is likely that this segment encoding the transporter acts as the stalk between the beta-barrel inserted into the membrane and the N-terminal head domain. The transmembrane domain analysis (www.cbs.dtu.dk/services/TMHMM) showed that the protein had no transmembrane and intracellular regions, so it was presumed as a secreted protein. This invasin gene was only found in *Salmonella enterica* serovar Tennessee strains and their closely-related isolates (Figure 5C). Meanwhile, the significant genetic linkage of the invasin and its neighboring genes was observed in these genomes.

**Figure 6:**
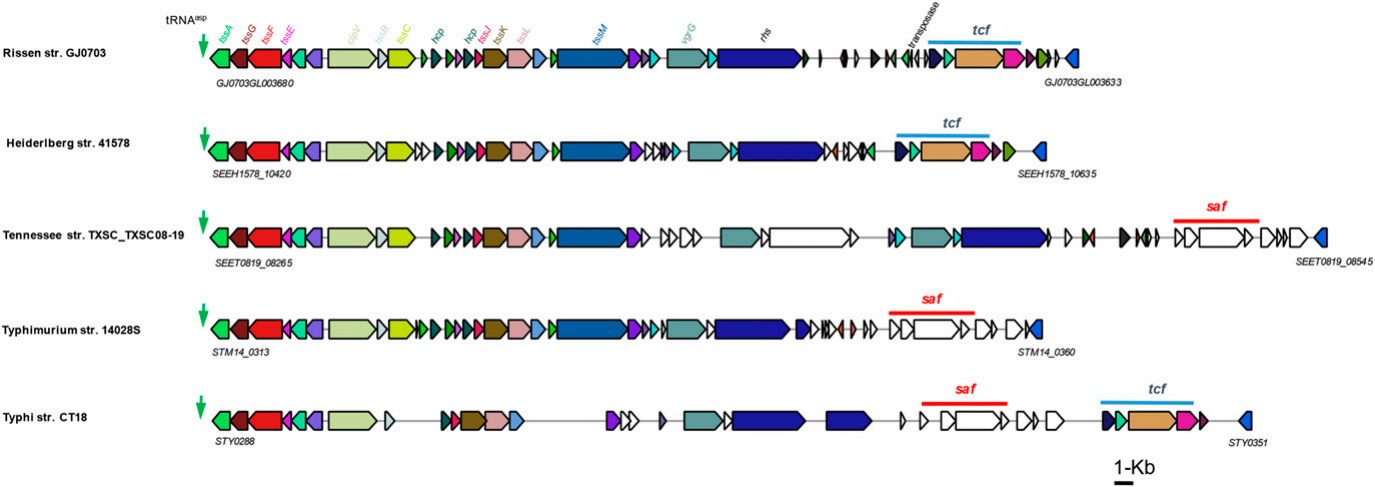
Schematic of the genetic organization of SPI-6 carrying T6SS. A genetic deletion involved the *saf*-associated gene cluster was found compared with strain CT18. The SPI-6 homologs of *S*. Rissen were collinearly compared with the counterparts in the genomes of strains Typhi CT18, Heidelberg 41578, Tennessee str. TXSC_TXSC08-19 and Typhimurium 14028S. The genes of mosaic SPI-6 homologs identified in a variety of *Salmonella* genomes were color coordinated, whereas specific genes and genome contexts were indicated by white.

SPI-6 is characterized by the presence of the T6SS gene locus, and S-fimbrial adhesin (*saf*) and Typhi colonization factor (*tcf*) operons. Compared with *S*. Typhi, most non-typhoidal *Salmonella* (NTS) serotypes do not possess the *tcf* operon, which encodes a Typhi colonization factor and plays a role in the host specificity of typhoidal serotypes. The *saf* operon encodes S-fimbrial adhesin, which is involved in bacterial pathogenesis. The genome analysis showed that strain GJ0703 harbors SPI-6, which contains a typical T6SS locus and *tcf* operon. However, the *saf* operon was absent in strain GJ0703 genome (Figure 6).

### Identification of virulence genes

To further identify virulence genes in *S*. Rissen strain GJ0703 genome, a BLASTp algorithm-based similarity search (identity > 75%, E value < 10-e5) was carried out using VFDB (mgc.ac.cn/VFs). Consequently, 119 virulence genes, including fimbrial adherence determinants, macrophage-inducible genes, magnesium-uptake genes, non-fimbrial adherence determinants, multiple T3SS effectors, a two-component system (PhoP–PhoQ), and colonization and invasion factors were identified. The dataset of virulence factors is displayed in Supplementary Table S6.

Owing to the apparent host preference of *S*. Rissen, the virulence factor profile of strain GJ0703 was compared with those of the broad-host-range serovar Typhimurium strain 14028S and the host-restricted serovar Typhi strain CT18. At present, 220 *Salmonella* virulence factors have been deposited in the VFDB, and strains 14028S and CT18 contain 150 and 169 known *Salmonella* virulence factors, respectively. In total, 108 of 220 virulence factors were shared by the three studied strains (Supplementary Fig. S7). In accordance to SPI-6 comparison, four virulence genes belonging to the *tcf* operon (*tcfABCD*) were shared by strains GJ0703 and CT18.

### Identification of antibiotic resistance genes and an integron-Like MDR region

Intriguingly, strain GJ0703 was observed to be resistant against a variety of antibiotics (listed in Supplementary Table S2). Antimicrobial susceptibility testing indicated that GJ0703 strain was resistant to tetracycline, ampicillin, sulfisoxazole and trimethoprim-sulfamethoxazole. We found a genomic region of plasticity encoding an integron accounting for multiple antibiotic resistance (MDR) traits in the GJ0703 genome via comparison on whole genomic scale against strain CT18, 14028S and closely-related serovar Tennessee str. TXSC_TXSC08-19 (Figure 4). A group of antibiotic resistant determinants were determined within this region, such as *sul3* and *tet(A)*. Meanwhile, numerous genes coding for insertion sequence elements, integrases and transposases were identified in the vicinity of drug resistance determinants, suggesting their on-going dissemination. In addition, *aac(6’)-Iaa* (*GJ0703GL002371*) was the only one antimicrobial resistance determinant observed to localize outside of this integron, which was proposed to confer strain GJ0703 aminoglycoside resistance.

**Figure 7:**
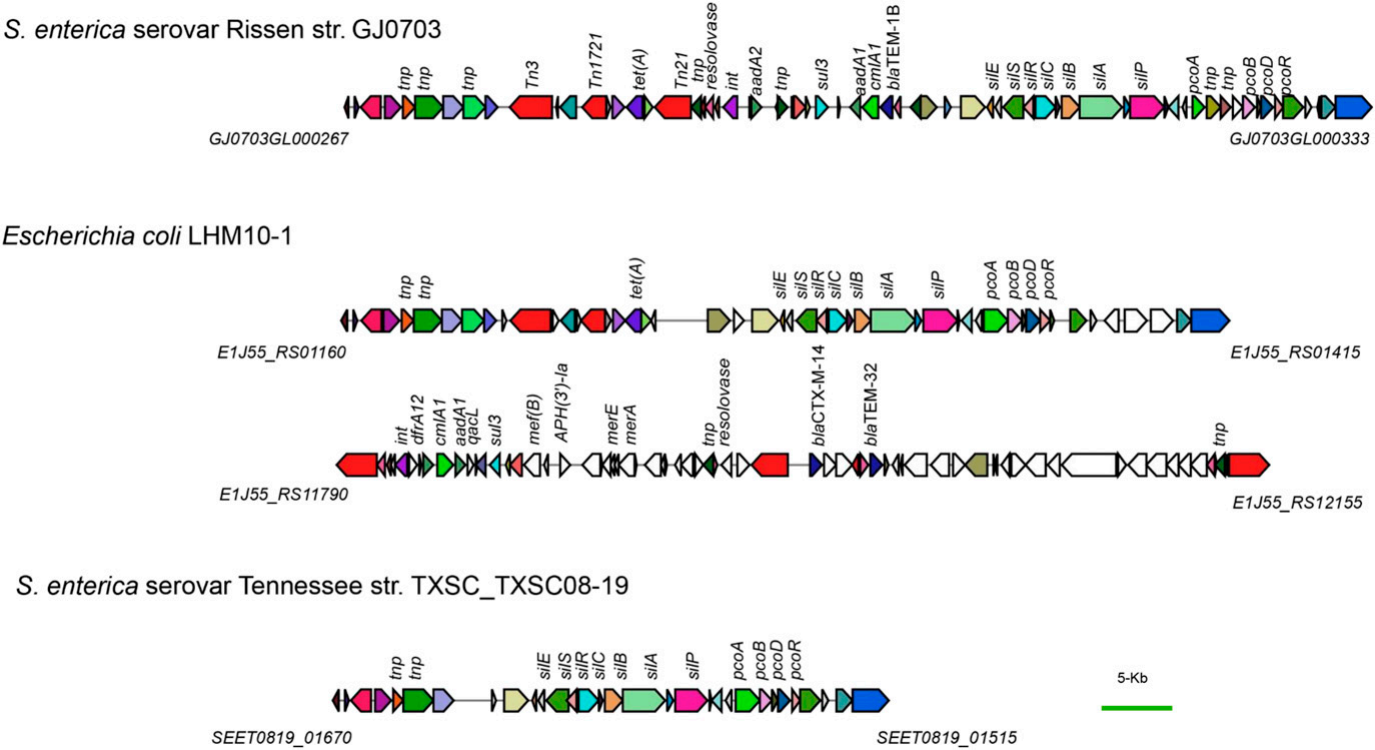
Schematic of the genetic organization of the identified integron-like region. Co-linear comparison of integron-like genomic regions was conducted for strain GJ0703 and strain TXSC_TXSC08-19 as well as *E. coli* LHM10-1. Tnp: transposase, Tn: transposon, int: integrase, Sul: sulfonamide-resistant dihydropteroate synthase, Tet: tetracycline efflux, QacL: chloramphenicol efflux, AadA: aminoglycoside nucleotidyltransferase, Bla: β-lactamase, Cml: chloramphenicol efflux, DfrA: trimethoprim-resistant dihydrofolate reductase, Sil: Cu^2+^/Ag^+^ efflux RND transporter, Pco: copper resistance, Mer: mercury resistance, Mef: macrolide efflux, APH: aminoglycoside O-phosphotransferase resistance to kanamycin.

By searching the available *Salmonella* genome, it was found that GJ0703 was the only strain with the intact architecture of this integron, even no homolog in closely-related serovar Tennessee and Rissen strains (Figure 7). Taken serovar Tennessee str. TXSC_TXSC08-19 for example, there are no gene predicted to be connected with a variety of heavy metal and antibiotic resistance in the likely ancestor of this integron.

Interestingly, the multiple antibiotic resistance related segment in strain *Escherichia coli* LHM10-1 was found as a homolog of this putative integron in GJ0703. However, there are several differences for their antibiotic resistance determinants as shown in Figure 7. Our finding indicated that GJ0703 had evolved into a genomic pattern with obvious abilities of infection and drug resistance. From this scenario, the GJ0703 progenitor could have suffered a diversified set of recombination and/or insertion to facilitate strain resistance capability transmission/spread. This characteristic prompted us to explore its possible foreign origin from horizontal transfer accompanied by the expanding size of the available bacterial genomes.

## Discussion

Virulence factors might contribute to the pathogenicity and host restriction of *Salmonella*, but the underlying mechanism is not yet fully understood. Although the genome sequences of several host-specific and broad-host *Salmonella* strains are available, the genetic basis involved in the host restriction of *Salmonella* remain unclear.

### Specific genes contribute to Salmonella host specificity

Gene gain or loss is generally associated with *Salmonella* virulence and host adaptation. For example, the expression of GtgE, a unique type III secretion system effector protein from the broad-host *S*. Typhimurium, allows *S*. Typhi to survive and replicate within mouse macrophages and tissues (Spano and Galan 2012). The earliest step in *Salmonella* pathogenesis is its attachment to the host cell, which is typically mediated by fimbriae that target particular cell types. The Tcf is a chaperone-usher fimbria, which may play a role in the host specificity of typhoidal serotypes. Although the *tcf* operon is present in at least 25 NTS serotypes, it contributes differently to the virulence of distinct NTS serotypes and exhibits a varying expression profile in different *Salmonella* serovars (Yue *et al.* 2012; Azriel *et al.* 2017). This operon is also observed in *S*. Rissen strain GJ0703 genome. Its expression profile and whether it contributes to intestinal colonization of *S*. Rissen need to be further clarified.

*Salmonella* adheres to intestinal epithelial cells using a myriad of fimbriae. Upon adhesion the subsequent uptake of *Salmonella* into mammalian cells is a complex process that is coordinated by a series of proteins. These proteins are normally effectors of T3SS encoded by SPI-1. These effectors have profound effects on the regulation of actin cytoskeletal structures and on mammalian cell-membrane plasticity. The net result of injecting these effectors into the host cell is bacterial-promoted endocytosis. *GJ0703GL000085*, located in SPI-3 of strain GJ0703 genome encodes a hypothetical toxin with 1149 amino acids. Although its function is unknown, it is similar to known invasins of *Salmonella*. The transmembrane domain analysis suggested that the protein was likely secreted and may function in helping bacteria to enter epithelial cells.

### nsSNPs of virulence factors may contribute to S. Rissen host specificity

In addition to horizontal gene transfer and genome degradation, pathoadaptive nsSNPs play an important role in the differential adaptive evolution of *Salmonella* (Guo *et al.* 2009; Kisiela *et al.* 2012; Yue *et al.* 2015). In the core genome, 3419 nsSNPs were found in strain GJ0703 and 1622 nsSNPs were shared by strains GJ0703 and CT18. The *mcpC* of strain GJ0703 harbors 18 nsSNPs, resulting in 16 amino acid mutations. McpC is a member of the methyl-accepting chemotaxis proteins (MCPs), which are transmembrane chemoreceptors for a vast array of environmental signals. Based on the sensory inputs of MCPs, chemotaxis is a key component in *Salmonella* virulence. McpC has two outside domains, two transmembrane domains and one inside domain. Most nsSNPs (14 of 16 amino acid mutations) were located in the ligand-binding sites of McpC’s inside domain, suggesting mutations of this domain may be involved in *Salmonella* virulence.

Iron is an essential cofactor for most bacterial species. The iron supply is normally limited in the environment because of the low solubility of Fe^3+^ at a neutral or basic pH level. To survive in an iron-deficient environment, bacteria have evolved the ability to biosynthesize dedicated small Fe^3+^-binding molecules, siderophores, to scavenge iron from the environment. The conversion of the enterobactin (Ent) siderophore to salmochelins, C-glucosylated Ent analogs, requires the *iroA* gene cluster, which includes five genes, *iroB*, *iroC*, *iroD*, *iroE*, and *iroN* (Bäumler *et al.* 1998). IroC is thought to be an inner membrane transporter, functioning in the export of apo-siderophores. IroE, thought to be periplasmic, converts Ent to salmochelins and hydrolyzes them as they are being exported out of the cell. IroC has 6 inside domains, 6 outside domains and 11 transmembrane domains. There are 17 nsNSPs in the *iroC* of strain GJ0703, resulting in 15 amino acid mutations. Most of these mutations (13/15) are concentrated at the C-terminal outside domain, where they may interact with IroE. IroE has one inside domain (1-12 aa), one transmembrane domain (13-30 aa) and one outside domain (31-305 aa). The four amino acid mutations in the IroE of strain GJ0703 are located at the C-terminal outside domain. Furthermore, enzyme activity assays revealed that the enzyme active site of IroE is located in the outside domain (Lin *et al.* 2005), suggesting that these nsSNPs may be involved in IroE activity.

Magnesium (Mg^2+^) plays a major role in biochemical functions, and Mg^2+^ depletion from phagosomes can be used by the host to limit the growth of intracellular pathogens (Blanc-Potard and Groisman 1997; Lavigne *et al.* 2005). MgtC is a critical virulence factor of intracellular pathogens that is involved in adaptation to low-Mg^2+^ environments and in adjusting the intracellular ATP level and pH value. A single amino acid residue substitution affects it stability and function (Lee *et al.* 2013; Choi *et al.* 2015). For example, W226 is involved in maintaining the stability of MgtC using FtsH-mediated proteolysis (Choi *et al.* 2015). However, the N92 mutation prevents MgtC binding to F1Fo ATP synthase and, consequently, abolishes the control of ATP levels and attenuates bacterial pathogenicity (Lee *et al.* 2013). E84 is essential for bacterial growth in a low-Mg^2+^ environment (Rang *et al.* 2007). There are two amino acid variations (141V and 145A) of MgtC shared by strains GJ0703 and CT18 compared with the other NTSs (Supplementary Fig. S8). These two residues are located in the cytoplasm of the fifth transmembrane domain, suggesting that these two amino acid variations may affect the function of MgtC and contribute to the host specificity of *S*. Rissen.

The earliest step in *Salmonella* pathogenesis is its attachment to the intestinal mucosa, which is typically mediated by fimbriae that targets particular cell types. Type 1 fimbriae are encoded by the *fim* gene cluster. Two nsSNPs in FimH (131S and 317N) and three in FimA (72D, 93T and 148A) are shared by strains GJ0703 and CT18. FimH is responsible for direct binding to oligomannosidic structures. FimA is important for attachment to enterocytes and promotes intestinal colonization of the host (Althouse *et al.* 2003). Amino acid replacements, resulting from SNPs throughout *fimH* confer different binding phenotypes. For example, the 223 position of FimH affects the host range of *S*. Typhimurium (Yue *et al.* 2015), FimH 158 amino acid residue affects bacterial binding to mammalian cells or to avian cells (Guo *et al.* 2009), and FimH 78I reduces the pathogenicity of *S*. Gallinarum and *S*. Pullorum to poultry. Therefore, nsSNPs of the *fim* operon might be involved in the host specificity of *S*. Rissen, but this needs to be confirmed through further studies.

In summary, *S*. Rissen is a widespread NTS and strain GJ0703 is flagged by a variety of genetic patterns accounting for its virulence and antibiotic resistance. The present study showed that *S*. Rissen GJ0703 has strong replication ability in human cells, and Tcf, AidA (GJ0703GL000085), McpC, the *iroA-E* gene cluster, type 1 fimbriae, MgtC mutations and other factors may be involved in the replication of *S*. Rissen in human cells (U937 cell line in this study). And an integron was predicted to provide antibiotics resistance capabilities for strain GJ0703, suggesting that GJ0703 has suffered distinct evolution compared with other Rissen isolates. These findings establish a foundation that will guide further studies on understanding of *Salmonella* host specificity.
